# What to expect? Injury patterns of Electric-Scooter accidents over a period of one year - A prospective monocentric study at a Level 1 Trauma Center

**DOI:** 10.1007/s00590-021-03014-z

**Published:** 2021-06-01

**Authors:** Andreas Harbrecht, Michael Hackl, Tim Leschinger, Stephan Uschok, Kilian Wegmann, Peer Eysel, Lars P. Müller

**Affiliations:** Faculty of Medicine and University Hospital, Center for Orthopedic and Trauma Surgery, University of Cologne, Kerpener Str. 62, 50937 Cologne, Germany

**Keywords:** E-scooter-related accidents, Injury patterns, Prevalence, Prospective, Monocentric

## Abstract

**Purpose:**

E-scooters are a new type of urban transportation utilized in Germany since June 2019, primarily in larger cities in the context of sharing offers. Such electrically operated standing scooters can be driven at a maximum speed of 20 km/h. A helmet is not mandatory. The aim of this prospective study is to document the injury patterns over the period of 1 year and compare our findings with already available data.

**Methods:**

Over a period of 1 year (July 2019–July 2020), data on E-scooter-related accidents treated at a level 1 trauma center of a major German city were prospectively documented. Injury patterns were analyzed, and epidemiological data evaluated.

**Results:**

Fifty-nine (35 female, 24 male) accidents were included in the observation period. Mean age of accident victims was 30.03 years (± 9.32). Alcohol influence at the time of accident was detected in 9 cases (15.25%). Many of the accident victims suffered multiple injuries. Most of the injuries were upper (50.84%) and lower extremity (47.45%) as well as craniofacial injuries (62.71%). A helmet was not worn in any of the cases.

**Conclusion:**

In the year following their introduction, E-scooter-related accidents have led to an additional burden on the emergency capacities of the involved University Hospital, especially in the summer months of 2019. Protective equipment is de facto not worn. Injuries to the extremities, head, and face were most common. None of the accident victims died. This coincides with results from other major cities in national and international comparison. A more intensive education about injury consequences of unprotected E-scooter use and the discussion of a possible obligation to wear a helmet and further protection equipment for the extremities should take place.

## Introduction

Since June 15th, 2019, E-scooters have been registered with road approval in Germany [1]. These are so-called electronically operated stand-up scooters. They have a maximum weight of 55 kg and maximum possible speed of 20 km/h. The scooters can be driven as early as age 12 with speeds limited to 12 km/h. At age 14, a maximum speed of 20 km/h is permitted. The limited maximum speed of 20 km/h also means that the use of the scooters does not fall within the scope of the helmet requirement.

The driving characteristics of the scooters are similar to those of a bicycle. Therefore, they may only be operated on bicycle paths, bicycle lanes, and if these are not available, on the road.

Alcohol limits are identical to those for driving a car. They are different from those for cyclists, as the electric motor of the E-scooter makes them a motor vehicle by definition. Mainstream availability of E-scooters is via sharing companies. There are around four major providers who distribute throughout the city of our clinic.

Initial police reports and press releases described an increased number of accidents, especially in the immediate summer months following the introduction of E-scooters in Germany. This was mainly due to the use out of curiosity about this new urban means of transport and a relevant driving insecurity [1].

Initial studies on injury patterns have already been published in many countries [2–6]. Injuries to the face and extremities account for the majority of these injuries. Most of accidents occur outside regular working hours, especially over the weekend, which places an additional burden on the emergency capacities of medical facilities [2].

The aim of this study is to present epidemiological data and analyze the injury patterns of E-scooter-related accidents treated at our clinic within 1 year. We hypothesize that due to the mechanism of the accident, facial and upper limb injuries occur predominately.

## Methods

This study is based on prospectively collected data on E-scooter-related accidents that were treated at our clinic in the period from 07/01/2019 to 07/01/2020. Inclusion criteria were all patients that suffered from E-scooter-related injuries and were 14 years or older (maximum speed: 20 km/h). Without exception, this only includes accidents that could be clearly identified as being caused using an E-scooter. Accidents caused by pedestrians or cyclists who were hit by an E-scooter were not recorded. In the large number of cases, this information could be collected in a standardized anamnesis interview in our emergency department by our trauma surgeons. In a few cases where the E-scooter users were brought to the emergency room with reduced vigilance, all necessary information could be obtained from the emergency doctors or paramedics who brought the patient to us. A trauma surgeon was always present in the emergency department and was responsible for treating the patients. Only cases that came to our emergency department primarily and were treated by us as first treatment were included. Presentations in our consultation hours due to follow-up treatment of E-scooter-related injuries that were first treated elsewhere were not included.

Gender, age, and date of accident were documented, as well as usage habits (helmet, self-inflicted accident, alcohol consumption), imaging (X-ray, CT/MRI), injury pattern, and treatment type (surgical or conservative treatment, inpatient or outpatient). Primary endpoint included all diagnosed injuries. Secondary endpoint included the rate of surgical treatment.

As the purely subjective findings about a possible alcohol consumption of E-scooter users on behalf of the treating medical staff were not permissible for this study¸ not every accident victim was subjected to laboratory sampling to investigate a possibly elevated ethanol level. Regarding this aspect, blood was only taken from patients in cases where alcohol blood testing became necessary due to a justified suspicion and the resulting duty of in-hospital care.

Patient data were pseudonymously and continuously collected during the period of 1 year.

A consultation with the ethics committee of the associated medical school was performed before acquisition of the data (20–1246). All human studies described were performed with the approval of the relevant ethics committee, in accordance with national law, and in accordance with the 1975 Declaration of Helsinki (current revised version).

### Statistics

All data was analyzed using SPSS (Version 20, IBM, Armonk, New York, USA) statistics software. Descriptive statistics were displayed. Number of cases in the various categories are also presented as percentages for illustration. Categorical variables were evaluated using the chi-square test. The level of significance was defined as a *p* value of < 0.05. The present study was designed according to the guidelines of Strengthening the Reporting of Observational Studies in Epidemiology (STROBE) [7].

## Results

In the observation period of 12 months 59 patients were included in this study. There were 35 female and 24 male patients. The mean age was 29 years for women (min: 17, max: 58, standard deviation (SD): 8) and 32 years for men (min: 19, max: 63, SD: 11).

Table 1 summarizes all basic and specific patient-related data.

**Table 1:** Basic data on documented E-scooter accidents at Cologne University Hospital within 1 year (*n* = 59)

	Total (%) *n* = 59	female (%) *n* = 35	male (%) *n* = 24	*p*-value
Blood alcohol	15.25% (9)	14.28% (5)	14.2% (4)	0.803
Self-inflicted	98.3% (58)	97.14% (34)	100% (24)	0.404
Helmet worn	0% (0)	0% (0)	0% (0)	-
Imaging performed	79.66% (47)	91.42% (32)	62.5% (15)	0.007*
X-ray	64.40% (38)	74.28% (26)	50% (12)	0.056
CT	32.20% (19)	37.14% (13)	25% (6)	0.327
MRI	5.08% (3)	5.71% (2)	4.17% (1)	0.79
Inpatient care	25.42% (15)	34.28% (12)	12.5% (3)	0.059
Surgical treatment	16.95% (10)	20% (7)	12.5% (3)	0.451

*Statistically significant difference (chi-square test)

A statistically significant difference between the sexes could be only be identified in the variable "imaging” (women in 91.42% of cases, men in 62.5% of cases, *p*-value = 0.007, Table 1).

In none of the documented accidents was a helmet worn. In only one case was a female E-scooter user injured through no fault of her own (Table 1). She was overlooked and hit by a car when turning. In the remaining cases (*n* = 58), the accident was self-inflicted.

General imaging (X-ray/CT/MRI) was performed in 47 cases (79.66%). A CT-scan was performed in 19 of those cases (32.20%), and 3 cases were examined by MRI (5.08%). In 12 cases (63.15%) where a CT-scan was indicated, it involved cranial imaging including the cervical spine. A complete body trauma scan was conducted in 3 cases (Table 1).

Surgical treatment became necessary in a total of 10 accidents (16.95%). In half of the surgical cases (*n* = 5) a midface fracture had to be treated by the Oral and Maxillofacial Surgery department. In the remaining cases, limb injuries were treated by the traumatology department involving reduction and osteosynthetic fixation (Table 2).

**Table 2:** Injury patterns and frequency of surgical treatment in E-scooter-related accidents treated within 1 year (*n* = 59)

Body part	Injury	Cases *n*	(%)	Surgical treatment *n* (%)
Head	Total	25	42.37%	0
	Intracranial bleeding	1		0
	Concussion	4	6.78%	–
	Contusion	10	16.95%	–
	Lacerations	10 *	16.95%	–
Face	Total	12	20.33%	5 (8.47%)
	Midface fracture	1 *		1 (1.69%)
	Nasal bone fracture	2 *		1 (1.69%)
	Lower jaw fracture	3 *		2 (3.39%)
	Anterior tooth trauma	10 *	16.95%	–
	Lacerations lip	5		–
Chest		0	0	0
Abdomen		0	0	0
Spine	Total	4	6.77%	0
	Cervical spine distortion	2		–
	Thoracic spine contusion	1		–
	Lumbar spine contusion	1		–
Upper extremity	Total	30	50.84%	2 (3.39%)
	Shoulder contusion	1		–
	Olecranon fracture	2		1 (1.69%)
	Radial head fracture	6	10.17%	0
	Elbow contusion	3		–
	Elbow ligament injury	1		0
	Distal radius fracture	2		0
	Wrist Contusion	10	16.95%	–
	Metacarpal fracture	1		1 (1.69%)
	Abrasions	4		–
Lower extremity	Total	28	47.45%	3 (5.08%)
	Hip contusion	1		–
	Knee contusion	10	16.95%	–
	Patella fracture	1		1 (1.69%)
	Proximal tibia fracture	1		1 (1.69%)
	Ankle fracture	1		1 (1.69%)
	Ankle distortion	5		–
	Metatarsal/toe fracture	2		0
	Foot contusion	3		–
	Abrasions	4		–

Selected injuries with indication of percentage; Some patients suffered from multiple injuries

* Treatment by oral maxillofacial surgery

Inpatient admissions were made in 15 cases (25.43%). In most cases (*n* = 10), an inpatient admission resulted from obligation to monitor after craniocerebral trauma. Most of the patients could be discharged without complications after 24 h. One patient suffered from a minor subarachnoid bleeding that occurred due to an unshielded head impact. The patient was monitored in the intensive care unit and finally treated without need for surgical trepanation. During the entire observation period, this patient remained the only case requiring intensive care (1.69%).

The blood alcohol level was measured legitimately in 9 cases (Table 1). The highest blood alcohol level of 2.2 per thousand was found in a 26-year-old and a 24-year-old woman. Both patients were monitored as inpatients.

**Fig. 1:**
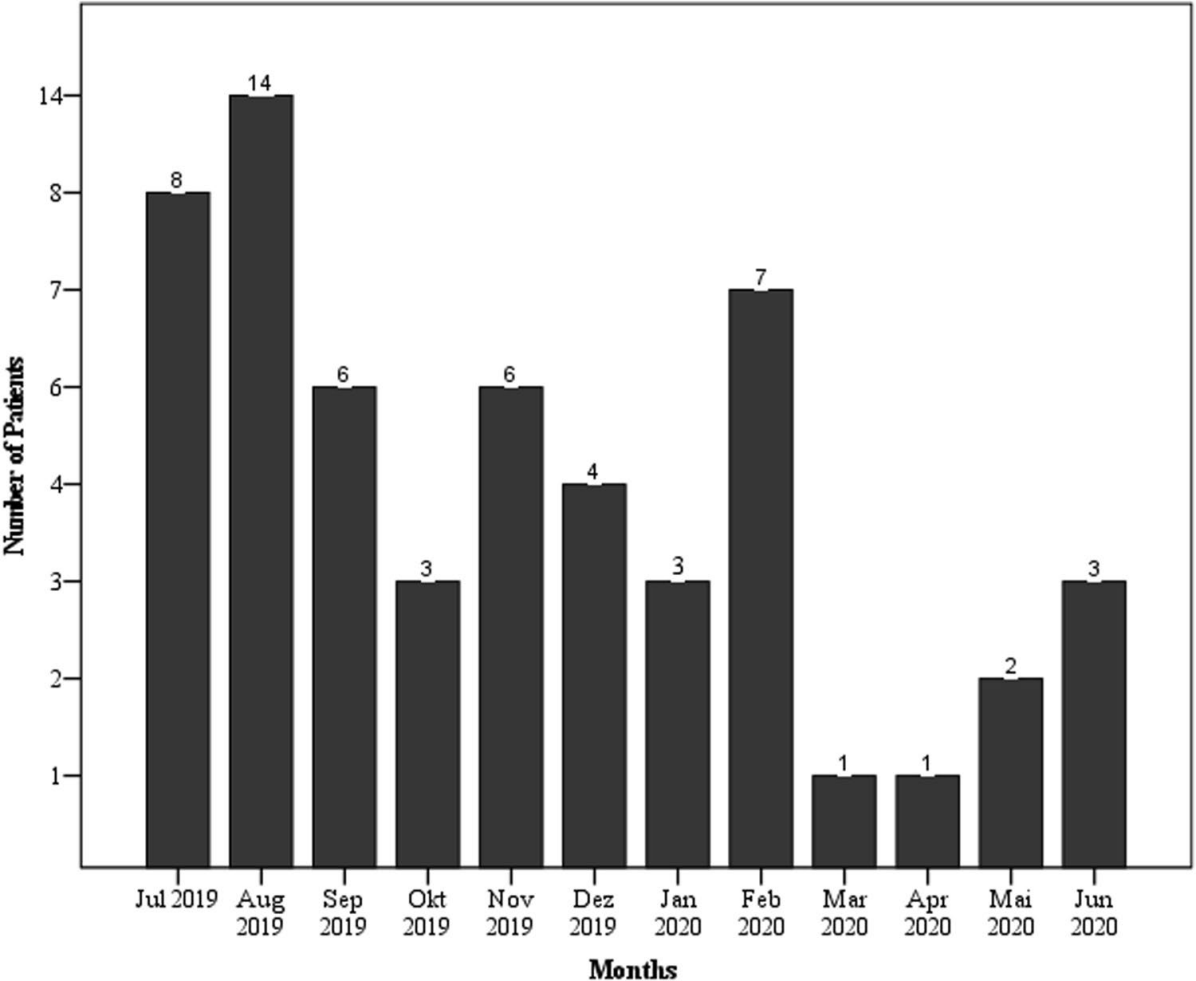
Monthly frequency of E-scooter-related accident presentations in the Central Emergency Department of the level 1 trauma center within 1 year

Most of the accidents occurred in the summer months of 2019 (*n* = 22, July–August), shortly after the introduction of E-scooters in Cologne. Figure 1 shows the distribution of cases over the entire year.

Table 2 summarizes injury patterns by individual anatomical regions. A total of 99 injuries were documented within 1 year. Several patients suffered from multiple injuries. The majority are injuries to the upper and lower extremities, as well as the head. Injuries of the upper extremity occurred in 50.84% (*n* = 30) of the cases, which in 2 cases resulted in the need for surgical treatment. They included a dislocated Mayo 2B olecranon fracture and a dislocated metacarpal 5 shaft fracture (Table 2).

The second most frequent injury was to the lower extremity (47.45%, *n* = 28). Non-severe injuries, such as contusions of knee joints, were very common (*n* = 10). Surgical treatment had to be performed in 3 cases (Table 2).

Finally, pure cranial injuries (42.37%, *n* = 25) constitute the third most frequent injury pattern of the examined patient collective with a large proportion of cranial contusions and lacerations (16,95% each, *n* = 25). 6.78% (*n* = 4) of accident victims suffered a concussion (Table 2).

## Discussion

This study describes the injury patterns of E-scooter-related accidents of a prospectively conducted investigation in a large German city over a period of 12 months.

E-scooters are a relatively fast means of transport characterized by a low center of gravity combined with small tires, a long steering axle, and a low drop height. In addition, there is a very strong braking effect and a short reaction time in case of an impending accident. Therefore, injuries to the head and upper extremities are frequent, as the fall can either still be absorbed by the hands or not.

In this study, the most common injuries were documented in the area of the head as cranial contusions without concussion symptoms (16.95%), in the area of the face as lacerations and anterior tooth traumas (16.95% each), in the area of the upper extremity as wrist contusions (16.95%) and radial head fractures (10.17%) and in the area of the lower extremity as knee contusions (16.95%, Table 2).

Abdominal injuries were not detected in any of the cases. This was also observed in other studies on E-scooter accidents [2, 8, 9]. Nevertheless, abdominal types of injury should always be considered, since handlebar impact into the abdomen, similar to bicycle riders, often cannot be excluded with certainty. Sonography of the abdomen should therefore be considered in these cases.

In the study with the largest number of cases to date, E-scooter-related accidents were investigated between September 2017 and 2018 in the Los Angeles metropolitan area at two trauma centers. A total of 249 associated accidents were treated in this region known as the birthplace of the E-scooter wave. Head injuries accounted for the largest proportion of injury patterns at 40.2%, with intracerebral hemorrhage in 2%. Fractures were the second most common injury (31.7%), with the upper extremity being the most frequently fractured [8].

There are two comparable studies about E-scooter-related accidents in a major German city. One is a study from Frankfurt, Germany, which documented E-scooter accidents prospectively from June 2019 to March 2020, and showed a similar overall picture. In 47.4% of cases, upper limb injuries and in 36.8% lower limb injuries were found. Head injuries occurred in 38.16% of cases [2]. This constellation is also reflected in our statistics with a slightly different weighting, thus underlining the hypothesis that such injuries are mostly to be expected in E-scooter-related accidents (Table 2). A study from Hamburg compared E-scooter accidents to bicycle accidents over a period of 1 year (June 2019–June 2020). They found in 89 E-scooter-related accidents that users more likely have accidents at night (37%,) and found them more likely to be under the influence of alcohol (28%). Facial injuries were documented in 54% of the cases [10].

We consider the greatest accident risk to be a relevant driving uncertainty of the users, which is since this type of transport was probably used for the first time. This was also described in the above-mentioned study from Frankfurt. In 32.9% of the cases, trauma occurred during the first use of an E-scooter [2]. However, as far as our study is concerned, data on this aspect have not been collected and are therefore merely an assumption.

In our study, the mean age of the E-Scooter users was 30 years. Comparable other studies show similar age distributions [1, 2, 8], which shows that this means of transport is particularly attractive to younger people [11]. Its use as a pure "joy-ride" should not be underestimated. Most accidents were documented during the summer months (37.29% of all accidents occurred in July and August 2019, Fig. 1). The reasons for this are twofold. On the one hand, the warm weather makes the ride very comfortable and on the other hand, the fact that the scooters were introduced in mid-June 2019 and thus represented a novelty in the field of urban transportation, provided a great incentive. This led to a predominant use out of curiosity and as a leisure opportunity. This is also reflected in the data from other countries [4, 12, 13]. This is also reflected in the increased use at weekends [2, 3, 8]. A study from California also describes in this context that E-scooters were used more by tourists, who often dispensed with safety protectors and helmets due to a spontaneous and short-term use [4].

Alcohol consumption represents an additional risk factor [10]. In 6.78% of all accidents (*n* = 4), the consumption of alcohol and the resulting accident led to severe head or facial injuries, which made inpatient admission for monitoring (subarachnoid bleeding, alcohol intoxication) or surgical treatment (multi-fragmentary mandibular fractures) necessary. In a study from San Diego, alcohol consumption was documented in 45.8% (*n* = 93) of 206 accidents [5]. We believe that the number of undetected cases is certainly higher in our study, since not every patient was routinely drawn blood for alcohol testing. The purely subjective findings on part of the treating medical staff about possible alcohol consumption by the accident victims were not permissible for this study. The publication of injury frequencies and severity of E-scooter accidents resulting from the use under alcohol influence may, however, contribute to the public education about the associated dangers [9].

Furthermore, the results of this study make it clear that safety concepts should be reconsidered. In international studies, the number of people wearing helmets is vanishingly small [8, 14]. However, concussions and serious head injuries could be reduced by wearing a helmet [5]. The protective properties of helmets are well known and described [15–17]. Similar to cycling, these protective properties are also transferable to E-scooter use and could lead to a reduction of serious head injuries. In a 2019 study, Allem and Majmundar, through a year-long investigation of picture posts linked to the official Instagram account of Bird (sharing provider of E-scooters), suggested that only 6.79% of users wore safety gear in the pictures [18].

Furthermore, the obligation to wear additional safety equipment could also be discussed. For example, skateboard or hoverboard accidents often show similar injuries to the upper extremity and/or head, as such recreational vehicles combine a low center of gravity with high speed, small wheels and a short reaction time in an urban setting as well [19–21]. Stoermann et al. presented the relevant comparison in this respect [2]. In 1997, Lewis et al. investigated the protective effect of wrist guards in a biomechanical study of human forearm specimens and found a significant increase in the kinetic energy required for a fracture compared to an unprotected wrist [22]. In our study, 22.03% (*n* = 13) suffered a wrist or hand injury that might have been less severe with protectors.

We detected more frequent indication for imaging in women (*p* = 0.007). We believe that the statistically significant difference in this aspect is due to the fact that men more frequently suffered from minor facial injuries such as anterior tooth trauma and cuts that did not require imaging. In 7 out of 9 cases where imaging was not required in males, the injury was a minor facial injury, whereas in 2 out of only 3 female cases where imaging was not required, the injury was a minor facial injury. The remaining female cases were more likely to be injuries to the extremities that required imaging. None of the patients included in this study died. There are also no international reports of fatal E-scooter-related accidents [2]. The SARS-CoV-19 pandemic caused a decrease in the use of E-scooters, likely contributing to a decrease in injuries related to E-Scooters requiring treatment at our hospital in March, April and May of 2020.

While E-scooter rental companies are promoting new safety concepts and educational campaigns regarding safety, the results of our study show that this has not yet led to success.

From a trauma surgeon’s perspective, we recommend a thorough evaluation of the neurological status of E-scooter victims to exclude intracranial hemorrhage. A CT should be performed in case of doubt. In addition, intraabdominal injuries should be ruled out by means of FAST (Focused Assessment with Sonography for Trauma) if the exact mechanism of trauma is unclear. Extremities should be examined for visible injuries and assessed by imaging. Additionally, oral and maxillofacial surgery expertise may be necessary. Young adults around 30 years of age with a high functional demand are to be expected.

### Limitations

This study is accompanied by limitations in interpretability. The fact that only one level 1 trauma center in the city was included, which incorporates a department for Oral and Maxillofacial Surgery, has two limitations. Firstly, the University Hospital has incomplete coverage of all patients that suffered from an E-Scooter accident in our city. Other and less serious traumas may not be included in the reported statistics, as they were treated by the surrounding level 1, 2 or 3 centers. Secondly, there is a possible clustered incidence of craniofacial injuries in our statistics, due to the expertise available.

Furthermore, the population density and composition as well as the availability of E-scooters and public transportation is different in every major city and results can thus not be transferred uncommented. Multicenter studies will most likely provide more information on exact frequencies of injury patterns and analyze the burdens on the health system in the future, further.

## Data Availability

Available from the corresponding author.
